# Separating early and intermediate vision EEG signatures of facial emotion recognition in a fast divided attention task to eye and mouth features

**DOI:** 10.3389/fcogn.2026.1871042

**Published:** 2026-08-05

**Authors:** Diana Costa, Camila Dias, Teresa Sousa, João Estiveira, João Castelhano, Verónica Figueiredo, Andreia Pereira, Miguel Castelo-Branco

**Affiliations:** 1 Coimbra Institute for Biomedical Imaging and Translational Research (CIBIT), University of Coimbra, Coimbra, Portugal; 2 Institute for Nuclear Sciences Applied to Health (ICNAS), University of Coimbra, Coimbra, Portugal; 3 Intelligent Systems Associate Laboratory (LASI), Guimarães, Portugal; 4 Institute of Physiology, Faculty of Medicine, University of Coimbra, Coimbra, Portugal

**Keywords:** EEG, face perception, facial expression, N170, P100, P250, social attention

## Abstract

**Introduction:**

Face emotion recognition is a core component of social cognition. The N170 event-related potential (ERP) is a well-known neural marker of face perception. However, the sensitivity of the N170 and other early and intermediate ERP components, such as the P100 and P250, to the emotional content of facial expressions remains debated, possibly due to the influence of attentional mechanisms. We investigated whether early facial emotion perception can be tracked by modulation of these EEG signatures when participants are required to selectively process both eye gaze and mouth emotional cues within a very short time frame.

**Methods:**

EEG data were recorded while 20 participants performed an emotion recognition task involving neutral, happy, and sad facial expressions. The task required the integration of gaze and expression cues within a brief 350-ms presentation, ensuring focused attentional deployment. We tested three hypotheses: (1) the P100 does not encode emotional expressions despite being modulated by attention; (2) the N170 is sensitive to emotional facial expressions; and (3) the P250 reflects higher-level emotional processing.

**Results:**

The P100 was not sensitive to facial emotional content. In contrast, both the amplitude and latency of the N170 were modulated by emotional expression, with larger amplitudes for happy compared with neutral and sad expressions, and longer latencies for happy and sad than for neutral faces. The P250 was also modulated by emotion, with sad faces eliciting lower amplitudes than happy and neutral faces.

**Discussion:**

These findings indicate that the P100 does not encode emotional face recognition when selective attention is controlled for. In contrast, emotional facial expressions elicit distinct N170 and P250 responses, supporting the involvement of early and intermediate neural systems in emotional face processing.

## 1 Introduction

Among socially relevant stimuli, facial cues and expressions play crucial roles in social cognition (Kuang, 2016; Salley and Colombo, 2016). They are particularly relevant in guiding individuals' attention toward socially meaningful stimuli, a process known as social orienting. The dynamics of facial emotion perception involve several neuronal circuits underlying structural face analysis and emotion recognition (Haxby et al., 2002). While studies using functional magnetic resonance imaging (fMRI) allow the identification of these circuits, the neuronal dynamics of face perception and their temporal signatures can be better studied based on electroencephalographic (EEG) signals (Nguyen and Cunnington, 2014; Bentin, 1996).

The N170 component is commonly used as a major neuronal marker of face recognition mechanisms. However, the literature is not unanimous regarding how the N170 is influenced by emotional content in facial expressions, and various paradigms have been used to investigate this question (Eimer et al., 2003; Rossion, 2014). Some of these paradigms include the Go/No-Go task (Aarts and Pourtois, 2012), face detection among objects (Pierce et al., 2011; Akbarfahimi et al., 2013), passive viewing (Wieser et al., 2012; Andreatta et al., 2012), emotional oddball tasks (Tsurusawa et al., 2008; Campanella et al., 2006), and facial emotions recognition (Chammat et al., 2010; Smith et al., 2013). The facial expressions most commonly examined include anger, fear, happiness, sadness, and disgust, with anger, fear, and happiness typically eliciting the largest amplitudes compared to neutral expressions (Utama et al., 2009). These results have been suggested to emerge from the need for rapid social responses to certain expressions (Hinojosa et al., 2015).

Structural encoding of faces is a multi-stage perceptual process. Its early stages involve fast feedforward extraction of facial cues (Thorpe et al., 1996; Crouzet et al., 2010) and the processing of individual features (Eimer, 2000). In contrast, later stages carry out configural analysis, that is, the spatial integration of these features into a coherent facial representation. It is possible to define different types of configural processing (first-order relations, spacing/relational processing, and holistic processing), and the integration of features into a configuration has been shown to be a later developmental process, distinct from basic feature detection (Maurer, 2002). Moreover, a meta-analysis of holistic and configural measures (composite effect, inversion) has provided evidence that feature integration is a distinct processing stage (Richler and Gauthier, 2014).

Event-related potentials (ERPs) provide a precise temporal window into the above-described stages of face processing. The P100, peaking around 100 ms over occipital regions, reflects early visual processing and attentional modulation but is not face-specific, responding primarily to low-level stimulus features (Bentin, 1996; Rossion and Jacques, 2011). In contrast, the N170, observed at 150–190 ms over lateral occipito-temporal sites, is considered the hallmark of face-sensitive processing, indexing the structural encoding of faces and demonstrating sensitivity to inversion effects (Bentin, 1996; Eimer, 2011). Accordingly, the P250 has been associated with the integration of facial features into a coherent configuration and with the early stages of identity recognition, reflecting more holistic processing and relying on recurrent interactions between visual areas (Itier and Taylor, 2004; Schweinberger and Neumann, 2016). Together, these ERP components delineate a hierarchical temporal sequence from early sensory processing to structural encoding and finally to integrative, identity-related face perception.

Structural encoding constructs a global facial representation and is indexed by the N170, which responds to head-like stimuli and salient internal facial features such as the eyes (Tso et al., 2018; Bossi et al., 2024). Configural processing, in turn, refers to the more detailed analysis of the spatial relationship among facial features (e.g., second-order relations such as eye-to-mouth spacing) and is reflected in N170's sensitivity to manipulations such as face inversion, which both delays the component and increases its amplitude (Maurer, 2002).

Some authors suggest that the N170 component arises from early automatic structural encoding of faces, occurring before structural cues comparison with representations stored in memory (Eimer et al., 2003; Ashley et al., 2004). However, others argue that structural encoding and emotion processing may be temporally distinct, as indicated by variations in N170 amplitude and latency in response to emotional expressions (Blau et al., 2007; Maratos et al., 2015). Prior findings have also suggested that the P100 component may be involved in face-specific visual processing (Dering and Donaldson, 2016; Monteiro et al., 2017). The P100 has been proposed to reflect an initial gist of a face, which facilitates subsequent identification of the physiognomic features processed at the configural level (N170) (Yang et al., 2020). However, the role of P100 in face recognition remains uncertain, as it is highly sensitive to attentional demands and can be modulated by selective, spatial, and non-spatial attention. Specifically, directing attention toward a stimulus significantly increases the amplitude of the P100, thus leaving its specific role in face recognition an open question (Dering and Donaldson, 2016). The P250 component is believed to be associated with higher-level aspects of face processing, reflecting the comparison between an incoming perceptual representation of a face and stored memory representations. Its amplitude is typically enhanced for familiar or repeated faces, indicating its role in identity recognition and face categorization (Dien et al., 2004; Resh Guptaa et al., 2019; Debruille et al., 2011). This component emerges after the initial structural and configural encoding (indexed by the N170), suggesting its involvement in more elaborate evaluative processes such as assessing familiarity, semantic associations, or emotional relevance (Schweinberger et al., 2002; Jemel et al., 2010). Puce et al. (2013) suggested that these ERPs form a robust positive–negative–positive (P100–N170–P250) complex involved in facial expression recognition and indexing early and intermediate stage visual (0–300 ms) processes involved in the encoding of the emotional face content.

Importantly, EEG provides temporal information about face perception that can be combined with source localization techniques to estimate the cortical origins of brain oscillations recorded at the scalp level. The inferior occipital gyrus (IOG), the lateral portion of the fusiform gyrus (FG), and the superior temporal sulcus (STS) have been described as the core system of face perception. IOG and FG are thought to mediate face encoding, while the STS is involved in processing social signals from faces, such as gaze direction and emotional expressions (Bernstein and Yovel, 2015; Freiwald et al., 2016). However, the relationship between the N170 and signals generated in these face-sensitive brain regions is still debated, with recent findings suggesting that the FG plays a significant role in the N170 ERP component (Nguyen and Cunnington, 2014; Jacques et al., 2019).

Our study aims to address such ongoing questions regarding the temporal dynamics and the electrophysiological nature of facial emotion perception. Specifically, we seek to investigate how early the emotional content in facial expressions modulates the response selectivity of P100, N170, and P250 ERP components. To achieve this goal, we employed a facial expression recognition task requiring focused divided attention within a very short time frame and analyzed these components at the amplitude and latency levels.

Attentional control was ensured by enforcing attentional focus on informative face features that needed to be used for subsequent task performance (eyes and mouth regions) thereby ensuring selective divided attention to specific features. Our study forced almost simultaneous attention to eyes mouth regions within a very short time frame of 350 ms, which requires very focused attentional deployment. Regarding other studies in the literature, Neath and Itier (2015) controlled for attention using a gender discrimination task. Schinkel et al. (2014) used target faces requiring either a gender decision (holistic task), or a decision on a facial feature (detailed analysis task). They suggested that the N170 adaptation profile can be modulated by top-down factors. Parkington and Itier (2018) used a similar framework as ours but featural attention was focused on single features, while in our study both eye and mouth regions were equally important. This is a critical feature of our study because it required divided featural attention and went beyond single featural attention. It allowed us to test prior claims on N170 modulation while differentiating them from P100 effects and critically testing the relevance of N250 modulation.

## 2 Materials and methods

### 2.1 Participants

Twenty adults with normal or corrected-to-normal vision and no history of neurological or psychiatric disorders were included in this study (nine females), mostly right-handed (19), and aged between 20 and 36 years (26.8 ± 4.5 years). The sample size was defined based on results of an “*a priori*” power analysis computed with GPower 3.1.9.7 [Parameters: effect size *f* = 0.35; α err prob = 0.05; power (1–β err prob) = 0.8]. The output of this analysis revealed that a sample size of 19 subjects is sufficient to evidence an interaction effect between the two experimental (facial expressions and ERPs) (Parkington and Itier, 2018). Written informed consent was obtained from all participants. The study was approved by the ethics committee of the Faculty of Medicine from the University of Coimbra. It was conducted in accordance with the principles of the Declaration of Helsinki.

### 2.2 Stimuli and task

**Figure 1:**
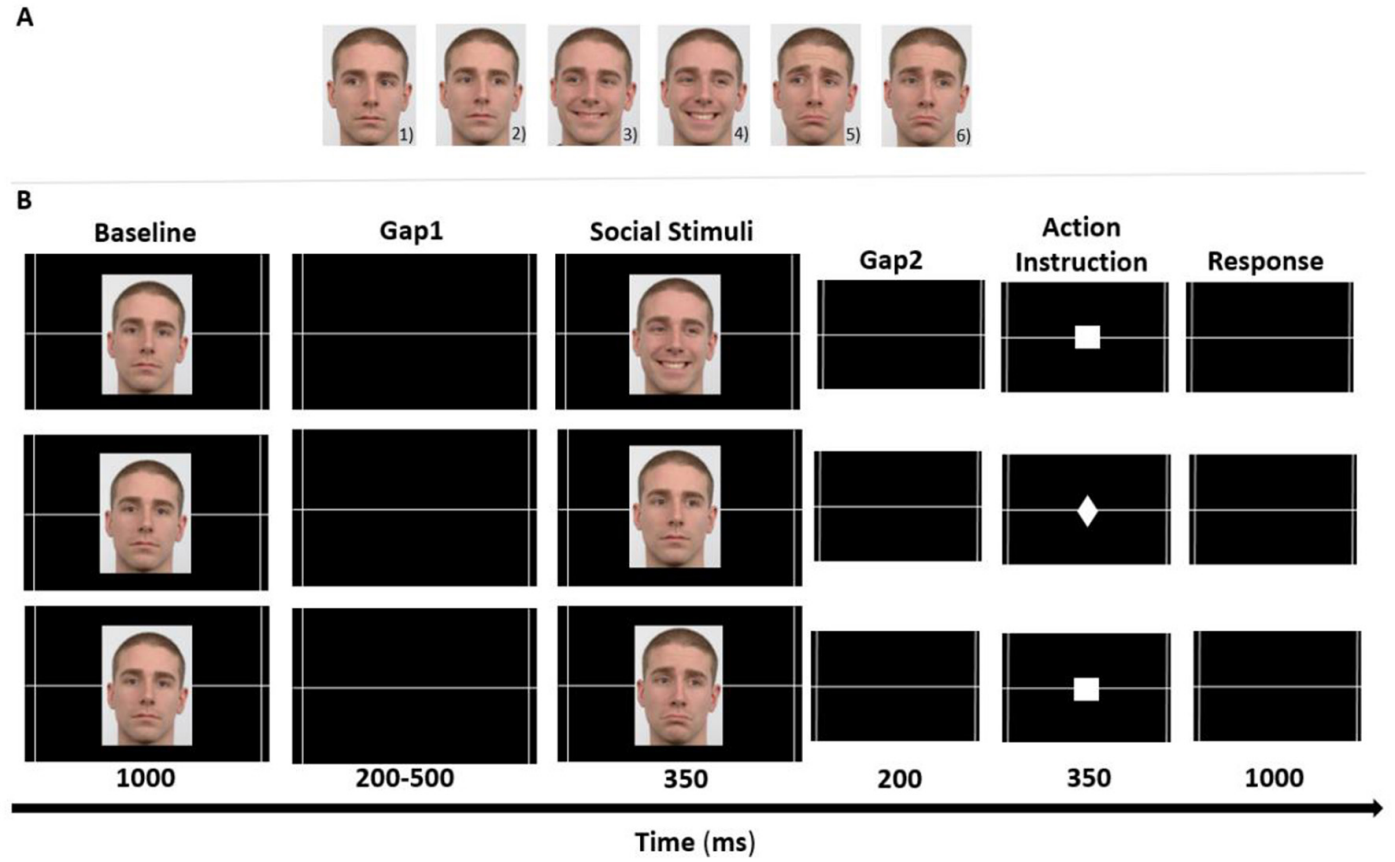
Overview of the facial stimuli paradigm used in our experiment. We used a go/no-go task based on six types of social stimuli **(A)** (1–6) with neutral, happy, and sad faces shown to the participants in a pseudo-randomized order. The paradigm included six stages **(B)** Baseline (neutral face, without any gaze type), Gap 1, social stimuli **(A)** (1–6) with right or left gaze, Gap 2, action instruction, and Response. Our analysis focused on the neuronal response to the social stimuli condition. Initially a neutral face was exhibited and followed by a Gap period. Then, a happy or sad face instructed participants to go and a neutral face instrutected them to no-go/take no action. Action was contingent on the gaze. After a gap period participants received a second instruction indicating the type of action to perform, which could be a saccade or a button press. A diamond indicated a saccade and a square indicated a button press to be given during the response period.

A standard set of color photographs depicting a Caucasian male with happy, sad, and neutral expressions were obtained from the Radboud Faces Database, which provides valence ratings, expression intensity, clarity, and genuineness (Lombardi et al., 2024). These images were displayed on a monitor with 1440 × 1080 pixels situated 60 cm away from the subject. The facial expression images had a mean luminance of 4.265 × 10^1^ cd/m^2^. Neutral, happy, and sad faces, each with a height of 9.02° and a width of 6.79°, were shown to the participants in a pseudo-randomized order (Figure 1A). The images were incorporated into a go/no-go task designed to study facial emotion processing and which enforced participants to track gaze and facial emotional features within a short time frame of 350 ms (Figure 1B). Each expression served as a cue for the participants to perform subsequent different actions of equal difficulty. The number of correct responses was compared across expressions to confirm that the amount of attention allocated was not dependent on the instruction provided attention allocation.

The facial expression used for the ERP analysis was presented for 350 ms as the instruction cue in a go/no-go task, following a baseline period in which a neutral face without gaze cues was displayed for 1000 ms, and a variable gap of 200–500 ms. A happy face cued to go and perform an action in the gaze direction of the eye gaze; a sad face cued to go and perform an action in the opposite direction of the eye gaze; a neutral face cue to a no-go/take no action. Upon visual stimulation, the participants were still unaware of what type of response (keypress or saccade) would be required. This was only known later. After a gap period of 200 ms participants received a second instruction for 350 ms indicating the type of response to give. A diamond with a height and width of 2.57° indicated a saccade and a square with a height and width of 1.82° indicated a button press. The response should be given during the following 1000 ms while seeing a black background. The white lines were displayed with intersections at a horizontal visual angle of 14.12° (measured from the center of the screen) to help homogenize saccade behavior.

This experimental paradigm was divided into 4 runs of 120 trials each (40 trials for each facial expression per trial). Keypress and saccade conditions were also balanced. The experiment lasted approximately 1 h, including the task explanation, setup preparation, and task execution.

### 2.3 Experimental setup and data recording

EEG data were recorded using a 64-channel cap with passive Ag/AgCl electrodes (Compumedics Quick cap; NeuroScan, USA). Participants' scalps were first cleaned with abrasive gel, and then the electrodes were placed on their head according to the international 10–20 standard system. Electrooculogram (EOG) data were recorded via two pairs of additional electrodes, placed above and below the left eye and in the external corner of both eyes. Reference channel was located between Cz and CPz, and electrodes impedance was kept under 20 kΩ during the recordings. The electrodes were connected directly to the SynAmps 2 amplifier system (Compumedics NeuroScan, Texas, USA) and sampled at 500 Hz. Data were recorded using the Curry Neuroimage 7.08 (NeuroScan, USA).

An eye-tracking system (EyeLink 1000 Plus from SR Research, Canada) was used to monitor saccadic responses. Data acquisition started with the nine-point calibration session, followed by a nine-point validation session of the eye tracker. The data were recorded at 1,000 Hz in a tower-mounted high accuracy (0.25°-0.5°) monocular eye-tracker. The recognition of saccadic movements was based on the horizontal coordinate of the point where the participant was looking at. The minimum distance from the display center and the minimum horizontal ocular movement amplitude to consider the existence of a saccade were of 4.66° of visual angle (measured from the center of the screen).

Keypress and saccadic trial information, including movement direction, response latency, and performance evaluation data, were registered using MATLAB files (^*^.mat), with keypress responses being recorded as soon as a key was pressed.

### 2.4 EEG processing

We used a MATLAB (R2018b) homemade script based on the EEGLAB toolbox functions for EEG signal processing and analysis. The EEG data were filtered using a Hamming windowed *sinc* finite impulse response (FIR) filter between 0.5 and 100 Hz. A notch filter was applied between 47.50 and 52.50 Hz.

Noisy channels were removed, and the remaining were re-referenced to the average of all EEG channels. Ocular, muscular, and cardiac artifacts were minimized based on independent components analysis (ICA) (Castelhano et al., 2014; Sousa et al., 2017; Makeig et al., 1995). Noisy components were identified and removed per participant. Channels previously removed were then interpolated using spherical interpolation. Data were segmented into epochs of 1,200 ms centered on the beginning of the social stimuli conditions, with a 200 ms [−200 0 ms] baseline period. Automatic epoch rejection was applied with an amplitude criterion of ±75 mV (with >80% of the trials accepted for further processing), followed by visual inspection to make sure only the noisiest epochs were removed (Castelhano et al., 2014).

### 2.5 Data analysis

#### 2.5.1 Event-related potentials (ERPs) identification

The N170 ERP component is characterized by a negative peak around 170 ms after the onset of a facial expression stimulus, most prominently observed at the lateral parieto-occipital and temporal electrodes (Calvo and Beltrán, 2014); whereas, P100 and P250 are positive components. The topology of P100 and P250 has also been described in the lateral parieto-occipital cortex, associated with rapid processes of selective attention (Nakamura, 2000; Amodio et al., 2014). In this study, we focused the analysis on a channel cluster, which yielded a regional weighted average of the electrodes showing the largest amplitude for the N170 component (TP7, P7, P5, PO7, T8, CP6, TP8, and P8). Similar clusters were previously tested and validated in face recognition paradigms (Langner et al., 2010; Ibanez et al., 2012; Bernardino et al., 2013). We conducted an additional analysis of the P100 component, focusing on occipital electrodes (O1, Oz, and O2), given that the occipital cortex constitutes the initial cortical stage of visual processing (Graewe et al., 2012; Gantiva et al., 2020).

Three ERP components were identified through the maximum (P100 and P250) and minimum (N170) peaks of each trial (Chammat et al., 2010; Kovarski et al., 2019). First P100 and N170 were identified within the time range of 50–200 ms, and the P250 within 200 to 300 ms. Peak detection for all components was performed using the MATLAB *findpeaks* function, ensuring consistent and automated identification of trial-level extrema. Then, the amplitude and latency of each ERP were estimated for happy, sad, and neutral faces for each participant by averaging the identified peak values per trial. As an additional validation of the main peak-based analysis, we performed a peak-to-peak measurement for the N170 and P250 components (P100–N170 and N170–P250 peak-to-peak amplitude). The temporal windows were derived from single-trial peak latencies and applied uniformly to ensure consistent and complete component capture across participants. Peak-to-peak amplitude was computed as the difference between the maximum and minimum values within each window, averaged across trials and channels.

Another supplementary analysis (Supplementary material, Figure 2) was conducted to compare faces vs. objects, in order to determine whether a different pattern of modulation could be observed between these categories, particularly in the P100 and N170 components. For this purpose, we analyzed 350 ms of the neutral face without gaze condition and compared it with the objects presented in action instruction condition.

**Figure 2:**
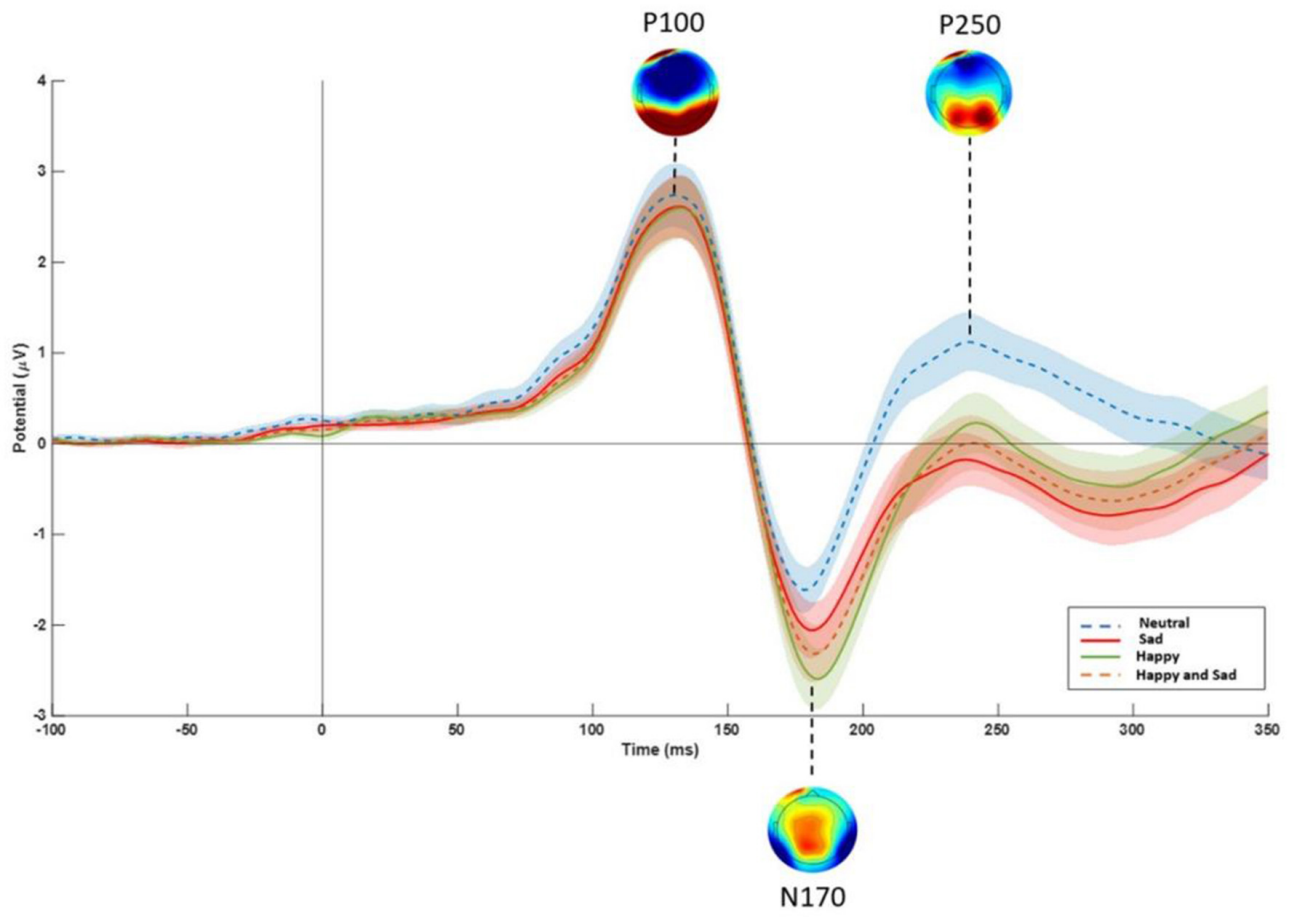
Early and intermediate ERP components during facial recognition of happy, sad, and neutral expressions. Grand-average waveforms for the P100, N170, and P250 components are shown. The results correspond to a temporoparietal channel cluster (TP7, P7, P5, PO7, T8, CP6, TP8, and P8). Responses to happy (green) and sad (red) expressions are also displayed together to illustrate their combined emotional pattern (brown dashed curve) relative to neutral faces (blue dashed curve). Shaded areas represent the standard error of the average waveform. Scalp topographies depict the voltage distribution for each ERP component (happy and sad expressions) at the mean latencies of 129 ms (P100), 181 ms (N170), and 245 ms (P250).

#### 2.5.2 Source estimation

Source localization was performed using standardized low-resolution brain electromagnetic tomography (sLORETA (version 2018); Pascual-Marqui). Preprocessed EEG data were first exported from MATLAB as single-trial epochs for each participant and experimental condition (emotional and neutral facial expressions).

Electrode positions were co-registered to a standardized anatomical template using fiducial landmarks and a standard 10–20 electrode montage. Source reconstruction was performed using a standardized head model constrained to cortical gray matter, aligned to the MNI (1988) atlas, with a spatial resolution of 5 mm.

For each participant and condition, current-density estimates were computed and averaged at the group level. Source current density was extracted for temporal windows corresponding to the N170 and P250 components; these windows were used exclusively for source-level quantification.

Voxel-wise paired comparisons of current density estimates between emotional (happy + sad) and neutral conditions were performed using Statistical Non-Parametric Mapping (SnPM) with 5000 random permutations. Family-wise error (FWE) correction for multiple comparisons was applied using the maximum-statistic method. Significant effects were defined as |t| > 5.367 (two-tailed, corrected *p* < 0.05).

#### 2.5.3 Statistical analysis

IBM SPSS Statistics 25 was used for running statistical analysis. For each ERP component (P100, N170, P250), a one-way repeated-measures ANOVA was conducted with facial expression (happy, sad, neutral) as the within-subject factor. These analyses were applied identically to both amplitude and latency measures. Additionally, we also run one-way repeated-measures ANOVA analyses to test the peak-to-peak amplitudes of the N170 and P250 differences per facial condition (happy, sad, and neutral). When the assumption of sphericity was violated, Greenhouse-Geisser corrections were applied. Peak and peak-to-peak measures were first extracted at the trial level and then averaged within each condition for each participant, resulting in one value per participant per condition before entering the ANOVA models. Throughout the study, differences were considered significant at *p* < 0.05 all the *post-hoc* comparions are Bonferroni corrected.

In addition to the ANOVA analyses described above, paired-samples *t*-tests were conducted to compare neural responses to faces vs. objects for each ERP component. These tests were applied to both amplitude and latency measures and were used to directly assess category-specific differences between the two stimulus types. All tests were two-tailed, and statistical significance was set at *p* < 0.05 (results in Supplementary material).

## 3 Results

### 3.1 P100, N170, and P250 response to emotional faces

To examine whether emotional facial expressions were modulated by ERP components, repeated-measures ANOVAs were conducted on the amplitude and latency of the P100, N170, and P250 across the three emotional conditions (neutral, sad, and happy; Figure 2).

ANOVA analyses comparing P100 amplitude and latency across emotional conditions revealed no significant differences. For amplitude, there was no main effect of emotional condition, *F*_(1.31, 24.80)_ = 1.10, *p* = 0.322, and η*p*^2^ = 0.055. Similarly, P100 latency did not differ across emotional expressions, the main effect of emotion on latency was not significant, *F*_(1.21, 22.96)_ = 0.72, *p* = 0.429, and η*p*^2^ = 0.037.

In contrast, the N170 showed clear emotional modulation in amplitude and latency. We found main effect of emotional condition in this component amplitude, *F*_(1.46, 27.76)_ = 13.48, *p* = 0.0003, and η*p*^2^ = 0.415. Bonferroni-corrected pairwise comparisons revealed that N170 amplitude was significantly more negative for happy faces (−2.168 ± 0.297) compared with neutral (−1.579 ± 0.149), *p* = 0.001), and sad faces (−2.168 ± 0.297), *p* = 0.003). In contrast, the sad-neutral comparison did not reach significance (*p* = 0.077). For latency, the emotional condition showed also a significant main effect, *F*_(1.42, 26.97)_ = 5.13, *p* = 0.021, and η*p*^2^ = 0.213. Pairwise comparisons showed that N170 latency was significantly shorter for neutral faces compared with sad *(p* = 0.004) and happy faces (*p* = 0.026).

For the P250, the main effect of emotional condition on amplitude was also found to be significant, *F*_(2, 38)_ = 6.67, *p* = 0.003, and η*p*^2^ = 0.260. Bonferroni-corrected comparisons showed that neutral faces (1.058 ± 0.384) elicited significantly larger amplitudes than sad faces (0.095 ± 0.274; *p* = 0.007), whereas neutral-happy (0.421 ± 0.261; *p* = 0.151) and sad-happy (*p* = 0.465) were not significant. In contrast, the main effect of P250 latency was not significant, *F*_(2, 38)_ = 1.33, *p* = 0.277, and η*p*^2^ = 0.065.

**Figure 3:**
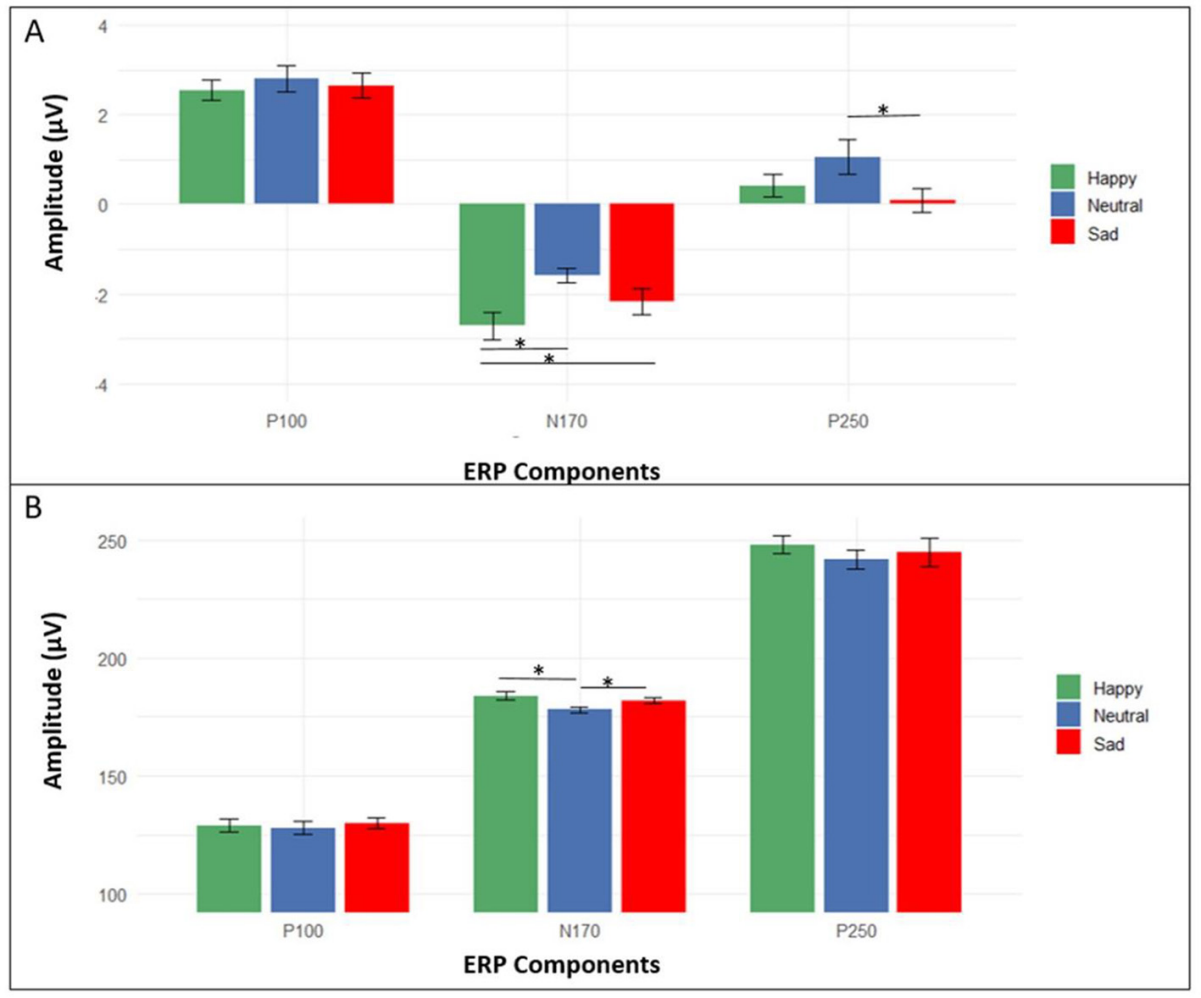
Mean amplitude **(A)** and latency **(B)** of the P100, N170, and P250 ERP components across emotional conditions (Happy, Neutral, and Sad). **(A)** Average amplitude (μV) for each ERP component. P100 amplitudes did not differ across emotional expressions. N170 amplitudes were significantly more negative for Happy faces compared with Neutral and Sad faces (*p < 0.05). P250 amplitudes were significantly larger for Happy compared with Sad faces. **(B)** Average latency (ms) for the same components. P100 and P250 latencies did not differ across conditions. N170 latency, however, was significantly shorter for Neutral faces compared with both Happy and Sad faces. Error bars represent the standard error of the mean (SE).

Figure 3 summarizes P100, N170, and P250 amplitude and latency differences for neutral faces and emotional (happy and sad) expressions. All amplitude and latency values (peak and peak-to-peak analyses) are detailed in Table 1. Complementary peak-to-peak analyses for the P100–N170 and N170–P250 components replicated a consistent modulation by emotional expression, with largerP100–N170 responses to happy faces and reduced N170–P250 responses to sad faces, reinforcing the patterns observed in the main ERP analyses. Full statistical details of these supplementary analyses are provided in the Supplementary material.

**Table 1:** Mean amplitude (μV) and latency (ms) values for the P100, N170, P250, and corresponding peak-to-peak measures across emotional conditions (Neutral, Sad, and Happy).

Parameters	Facial Expression	P100	N170	P100–N170 (peak to peak)	P250	N170–P250 (peak-to-peak)
Amplitude	Neutral	2.81 ± 0.29 μV	−1.578 ± 0.15 μV	5.72 ± 0.43 μV	1.06 ± 0.38 μV	3.45 ± 0.31 μV
	Sad	2.65 ± 0.28 μV	−2.17 ± 0.30 μV	5.20 ± 0.44 μV	0.10 ± 0.27 μV	3.09 ± 0.28 μV
	Happy	2.55 ± 0.22 μV	−2.70 ± 0.30 μV	4.86 ± 0.40 μV	0.42 ± 0.26 μV	3.62 ± 0.26 μV
Latency	Neutral	128 ± 2.64 ms	178 ± 1.26 ms		242 ± 4.06 ms	
	Sad	130 ± 2.34 ms	182 ± 1.18 ms		245 ± 6.07 ms	
	Happy	129 ± 2.06 ms	184 ± 1.94 ms		248 ± 3.94 ms	

Supplementary analysis in the P100 occipital channels (O1, Oz, and O2) showed that both amplitude and latency did not differ between emotional and neutral faces, indicating that early occipital processing was not modulated by facial expression.

To ensure that participants paid equal attention to all tested conditions, we analyzed the number of correct and incorrect responses associated with each facial expression used as an action cue. Each run contained a total of 120 trials, equally distributed across the emotional conditions. The analysis revealed 2,452 correct actions and 243 errors for happy expressions, and 2,428 correct actions and 282 errors for sad expressions. A *t*-test comparing the error rates between the two expressions, *t*_(19)_ = 1.600, *p* = 0.126, indicated no significant difference. These findings confirm that the results are not influenced by differences in error rate.

### 3.2 Source estimation for the ERP components

**Figure 4:**
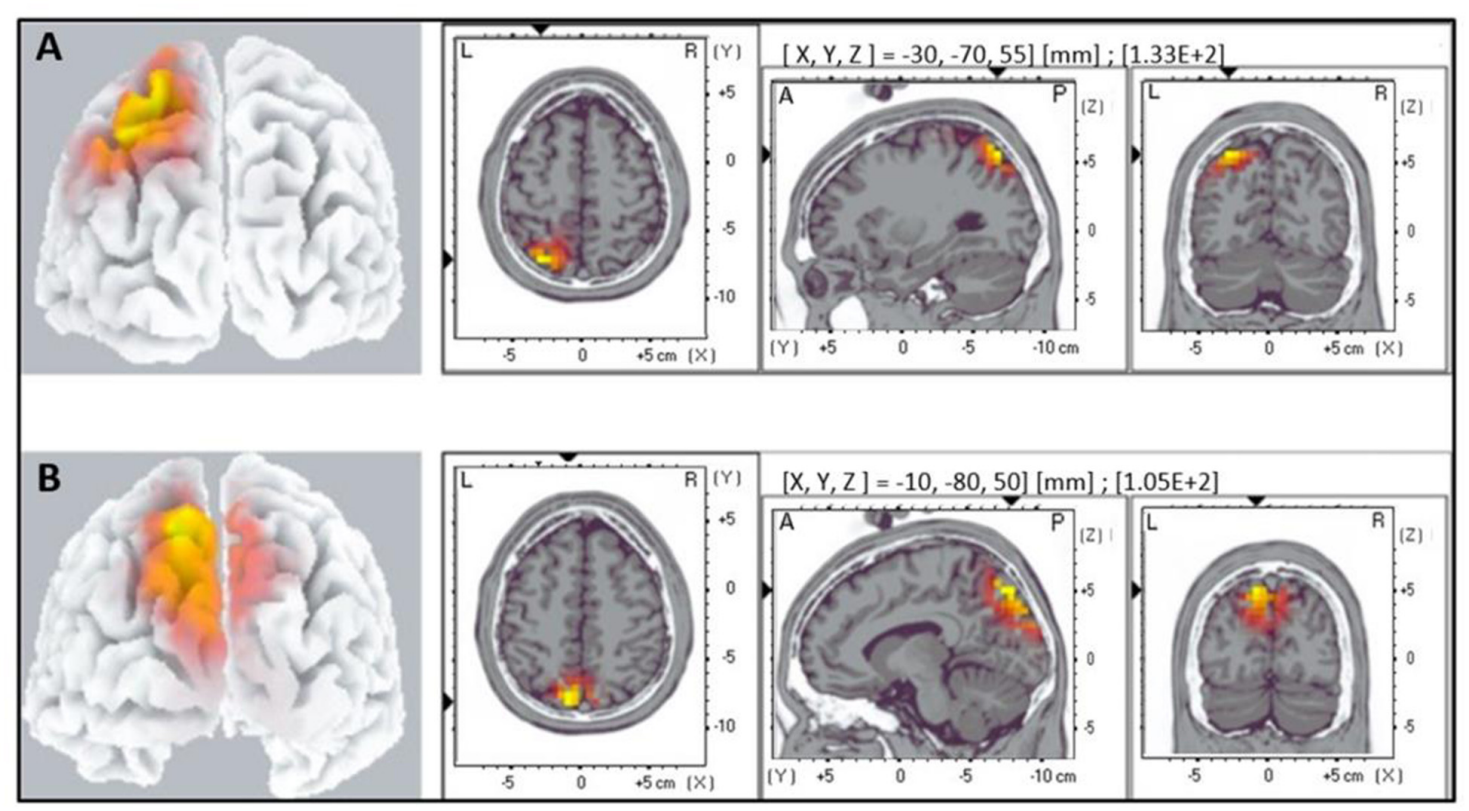
Statistical maps showing source localization significant emotional-neutral differences for the N170 and P250 components. Warm colors indicate regions where estimated cortical current density was significantly greater for the emotion than the neutral condition for each ERP. **(A)** N170 source estimated in the parietal lobe, BA7 [*x* = −30, *y* = −70, and *z* = 55]. **(B)** P250 source estimated in the parietal lobe/BA7, precuneus [*x* = −10, *y* = −80, and *z* = 50].

Source estimation analysis (Figure 4) was performed on ERP difference between emotional and neutral conditions for the N170 and P250 components, as these ERPs showed modulation in response to facial emotional expressions. For the N170, the maximum difference peaked in the parietal lobule, in proximity to BA 7 (MNI coordinates: *x* = −30, *y* = −70, and *z* = 55; *p*= 0.0002). The P250 comparison between emotional and neutral faces revealed maximal source estimates at MNI coordinates: *x* = −10, *y* = −80, and *z* = 50, corresponding to the precuneus (BA 7, *p* = 0.0002), also compatible with involvement of medial parietal regions.

Overall, the observed patterns in source space were predominantly localized within parietal cortical regions associated with visuospatial and social-cognitive processing (Kawashima et al., 1995; Kamali et al., 2014).

## 4 Discussion

In this study, we explored the temporal dynamics of the neural mechanisms involved in emotional face recognition, with a particular focus on the modulation of not only the N170 but also other related components like the P100 and P250 by the emotional content of facial stimuli. Our study employed a face recognition task while ensuring attentional focus by forcing fast divided attention between eye and mouth features within the very short time span of 350 ms, aiming to identify amplitude and latency differences between neutral and emotional expressions, specifically happy and sad faces. Additionally, we sought to investigate the neural sources associated with facial emotion recognition.

The N170 is a well-established ERP marker of face processing (Kuang, 2016). However, there is ongoing debate regarding its selectivity for emotional content in facial expressions. Some studies support the idea that emotional facial expressions influence N170 amplitude (Utama et al., 2009; Kamali et al., 2014; Li et al., 2008), while others suggest that this low-level ERP component is primarily modulated by basic features of facial stimuli, such as the wide-open eyes and increased size of the white sclera around the dark pupil in fearful faces, rather than discriminating between expressions (Chammat et al., 2010; Luo et al., 2010). Our findings contribute to this debate by demonstrating the presence of an emotional effect when attention is focused on dual facial features. We found a significant difference between facial expressions for the N170 amplitude. The results revealed an N170 amplitude distinction between happy and sad faces, a finding less commonly reported in the literature (Eimer and Holmes, 2007). By calculating the total error associated with happy and sad expressions, we confirmed that the differentiation in recognition between these emotions occurs regardless of the action instruction associated with each of them. In other words, the ERP modulation recorded was independent of the number of errors and the type of action.

It is also important to clarify that within our paradigm, that both *go* and *no-go* stimulus cues imply attentional load. Stopping is an active control process, not merely absence of action and that inhibition competes with response execution in prepotent action cancellation. The *go* response constitutes a prepotent alternative, due to the high frequency of *go* trials and greater practice of that response. A *no-go* cue entails executive load because the participant must inhibit possible go responses, which are more frequent. In such cases, the participant must detect the *no-go* stimulus and inhibit a response that is already partially prepared. Here, attentional or executive-control load is similar, because the participant has to detect the *no-go* cue and suppress a prepotent response. Foundational models of response inhibition show that stopping is an active control process, not merely the absence of action (Logan et al., 1984; Logan and Cowan, 1984). When the go response constitutes a prepotent action, due to its high frequency, greater practice, or the initial activation of a motor tendency, the occurrence of a *no-go* stimulus may require equal or even greater levels of executive control. In these circumstances, the participant must detect the no-go stimulus and inhibit a response, a process that involves active stimulus monitoring and maintenance of the task rule (Verbruggen et al., 2014; Wessel, 2018).

Moreover, the fact that the stimulus-response mapping is presented only after the visual stimulation further reinforces that cognitive load is not reduced. In the absence of the mapping, specific motor preparation is constrained, particularly under divided-attention conditions, requiring the participant to (1) encode the visual cues, (2) maintain it temporarily while distributing attention (face and mouth) and (3) apply the delayed rule only later. This is consistent with evidence that perceptual processing, response selection, and motor preparation are dissociable stages, and that incomplete or missing information delays final motor specification (Miller and Low, 2001; Rosenbaum, 1980). Additionally, dual-task research demonstrates that response selection under divided attention imposes a central bottleneck, increasing cognitive demands (Pashler, 1994). Thus, 350 ms are required to distribute divided attention which does not allow for full preparation of a specific motor command, which is anyway delayed.

Taken together, these considerations indicate that no-go stimuli may impose inhibitory control demands comparable to or greater than those of go trials, particularly in contexts where the go response is dominant or expected. This interpretation aligns with electrophysiological evidence showing that no-go effects are modulated by conflict and trial-type frequency, rather than by withholding alone (Nieuwenhuis and Yeung, 2003). The delayed mapping in our paradigm therefore prevents any meaningful reduction in cognitive load.

N170 has been proposed to represent early configural stages of face processing, which may reflect an activation related to the structural coding of faces, and from this point of view response levels might be expected to be relatively invariant to emotional details in facial expressions (Crouzet et al., 2010; Chen et al., 2014). However, Li et al. (2008) proposed that the period from 150 to 300 ms constitutes a second stage of facial expression processing, already sensitive to emotion features in general. The authors argue that distinction between emotions specifically occur only in a third stage, which can begin around 300 ms (280–450 ms). Our results are, therefore, in line with this hypothesis, suggesting, however, that the distinction between expressions may occur in this second stage, where we were able to observe a significant distinction between happy and sad expressions. Chamberland et al. (2017), in an attentional perception study, also observed the differentiation between fear and surprise emotions in this second stage (N170) when attention was directed to the mouth area.

Regarding N170 latency, our results did not show differences between emotional (happy vs. sad) expressions but did reveal distinctions between neutral faces and happy or sad faces. This latency pattern, where neutral faces are processed more quickly than happy and sad faces, is at odds with the notion that emotional faces, especially those conveying strong emotions like happiness or sadness, could potentially be processed more rapidly due to their higher salience and evolutionary relevance, as emotional content tends to capture selective attention earlier in processing (Blau et al., 2007). Through the modulation of facial expressions, the emotional effects of the N170 are more frequent in amplitude (Hinojosa et al., 2015). However, our latency results reinforce the hypothesis that there is an emotional differentiation at the level of the N170 component.

P100, the earliest stage of visual processing, has been associated with low-level characteristics of a visual stimulus and thus hypothesized to be less likely modulated by the content of facial expressions (Li et al., 2008). While some studies suggest its potential differentiation between expressions, such as fear and anger or happiness and anger (Logan and Cowan, 1984; Verbruggen et al., 2014), we did not find significant differences in P100 amplitude and latency between happy, sad, and neutral expressions. This is in line with other previous studies suggesting that this ERP component is not significantly modulated by emotional expressions (Wessel, 2018). It has also been described that the P100 can also be modulated by attention (Dering and Donaldson, 2016). Therefore, our results suggesting no significant effect of emotional cues on P100 when controlling for attention, are in line with previous evidence that negative and positive expressions cannot be reliably differentiated as early as by the P100 component (Smith et al., 2013; Logan and Cowan, 1984). Moreover, the P100 is also believed to play a significant role in face discrimination from other object types, showing amplitude differences between faces and non-faces (Miller and Low, 2001; Rosenbaum, 1980; Pashler, 1994). In our study, we conducted a supplementary analysis (Figure 2 in the Supplementary material) in which we examined the P100 and N170 components in the detection of faces and objects (squares or diamonds). For the P100, the amplitude for faces was indeed higher than for objects, but this difference was not significant, with a clear distinction observed only in the N170 component.

Regarding the P250 component, which has been described to be part of the P100–N170–P250 complex (Yang et al., 2020) its role in facial emotion recognition remains a subject of debate. Some studies suggest that P250 is responsive to emotional expressions, with increased amplitude for emotional expressions compared to neutral (Nieuwenhuis and Yeung, 2003), while others found a reduction in amplitude for happy and sad faces when compared to neutral faces (Kovarski et al., 2019). Two main hypotheses are described in the literature to explain this reduction. One hypothesis suggests that the reduction in amplitude may be attributed to a redistribution of attentional resources. This results in a decreased amplitude for emotional expressions and enhances the distinctions between different emotion categories. The second hypothesis proposes that the lower P250 amplitudes for negative emotional expressions may indicate a facilitated access to negative emotions compared to neutral or positive ones. This may be the result of evolutionary survival processes, where negative emotions require faster recognition for adaptive responses (Batty and Taylor, 2003). These two hypotheses are in line with our results, emotional expressions have a lower amplitude compared to the neutral expression, with the sad expression showing the lowest amplitude and a significant difference compared to the neutral face.

As both the N170 and P250 components were modulated by the experimental conditions, source analysis was conducted to estimate the cortical regions that may have contributed to the observed ERP differences. For the N170 component, source estimation suggested maximal difference peaking at the dorsal parietal region, consistent with the involvement of the superior parietal lobule and other dorsal regions implicated in visuospatial attention and social cue integration (Chamberland et al., 2017; Chiao et al., 2008).

This interpretation is consistent with the task demands, as participants were required to process gaze direction and facial expressions simultaneously, an integration thought to recruit dorsal visuospatial and attention-related networks. Previous research has shown that the superior parietal lobule plays an important role in visuospatial attention, the integration of dynamic social information, and the allocation of attentional resources to socially relevant stimuli (Klucharev and Sams, 2004; Morel et al., 2009). In the context of face perception, it may contribute to higher-order processes such as orienting attention to salient facial features and integrating gaze direction with emotional meaning (Chiao et al., 2008; Goffaux et al., 2003). Thus, the dorsal parietal contribution observed here may reflect the integration of spatial and emotional information during social perception.

In this study, the P250 was estimated to originate in the precuneus. This region is believed to be involved in visuospatial processing, directing attention in space, and perspective-taking, as part of the Dorsal Attention Network, Ventral Attention Network, and Frontoparietal Network, both from cognitive and affective perspectives (Dering et al., 2009, 2011). However, another study by daSilva et al. (2016) found a source of this ERP in the right fusiform gyrus during a simple visual search task, with greater current density observed for fear compared to disgust conditions. In another study, Simões et al. (2018), using a facial morphing visualization and an imagery task, source localization revealed significant results in the precuneus, as we also observed in our study. The fusiform gyrus is strongly involved in face recognition, an important component in emotional recognition tasks (Chang et al., 2010).

Converging evidence suggests that the superior parietal lobule and the precuneus cooperate in directing selective attention in the absence of overt motor responses (Cavanna and Trimble, 2006). In a Simon et al. (2002) study, participants performed various tasks, including attention, pointing, grabbing, saccades, and calculating. Both brain regions were activated by the saccadic and pointing task, while the precuneus showed more activation only for selective attention. Le et al. (1998) reported that the shift of attention to visual stimuli, when compared to sustained attention, produced bilateral activation in these both regions. This spatial analysis contributes to the understanding of how neural resources are allocated during the perception of emotional faces (Kuang, 2016).

Although attentional control tasks inevitably involve some degree of executive processing, our paradigm was specifically designed to minimize potential confounds related to motor execution, as the instructions for performing the motor responses were presented only after the visual stimulation. The analysis of participants performance revealed no significant difference in error rates between happy and sad facial expressions, indicating that emotional valence did not differentially impact task performance. Moreover, to assess whether the type of motor response influenced emotional effects, in our prior work Estiveira et al. (2022), we implemented two distinct action mappings, saccadic and button-press responses, tested across participants. In these study, the comparison between two emotions (happy and sad) and the two action mappings (button press and saccadic) revealed no significant main effect of action mapping. These results demonstrated that the main emotional effects at the neural level were not driven by differences in response modality. Additionally, the eye-tracking data confirmed that spatial attention was effectively and similarly allocated across all experimental conditions, as no significant differences were observed in fixation duration or distribution across facial expressions. Thus, neither motor demands nor spatial attention differences can account for the ERP modulations observed. The engagement of regions such as the superior parietal lobule and precuneus is therefore better interpreted in light of their established roles in emotion-guided attention and socio-cognitive processes.

This work contributes to the ongoing discussion of ERP modulation by facial expressions, particularly focusing on the N170 and P250 components. In addition to differences in recognition between neutral and emotional expressions, our observations also highlight distinctions between positive (happy) and negative (sad) expressions. The findings further contribute to the debate on whether the N170 component can serve as a biomarker in conditions involving socio-communicative dysfunction, especially in the domain of facial processing.

One important limitation of the present study concerns the restricted variability of facial stimuli. Specifically, only a single male Caucasian model from the Radboud Faces Database was used across all experimental conditions. This methodological choice limits the generalizability of the findings, as ERP responses to emotional faces may vary according to stimulus characteristics such as facial identity and gender. Future studies should include a more diverse set of face stimuli, incorporating multiple identities with variation in gender to improve external validity and assess the robustness of the reported effects across different facial characteristics

In summary, our study provides evidence for the temporal modulation of the N170 component, with emotional content influencing both its amplitude and latency, when selective attention is focused simultaneously on dual eye and mouth features with a short time window of 350 ms. Additionally, we demonstrate that the P250 component may also be modulated by emotional expressions. This suggests that the differentiation of emotions is not confined to the N170 but may extend into the later P250 stage. However, the P100 component appears less affected by emotional expressions, consistent with its non-selective role in emotional face recognition, aligning with its function in early sensory processing. Our paradigm, which controls or non-specific attentional factors, reinforces the idea that emotional differentiation occurs during early ERP stages, specifically at the N170 and P250 levels.

Furthermore, our findings align with previous studies on the neural correlates of emotional face perception under focused attention, providing new insights into the involvement of early ERP components in processing facial emotions.

## Data Availability

The raw data supporting the conclusions of this article will be made available by the authors, without undue reservation.
