# Supplementary material for: Separating early and intermediate vision EEG signatures of facial emotion recognition in a fast divided attention task to eye and mouth features
Source: Front Cognit. 2026 Aug 5;5:1871042. doi: 10.3389/fcogn.2026.1871042 (PMC13485748; doi:10.3389/fcogn.2026.1871042)
Supplement: Supplementary file 1 [file Data_Sheet_1.docx]

**Supplementary Analysis**

**Peak‑to‑Peak Amplitude of N170 and P250**

**Supplementary Figure 1**: Peak‑to‑peak amplitudes for the P100-N170 and N170-P250 ERP intervals across emotional conditions (Happy, Neutral, and Sad). The bars represent the mean amplitude (µV) for each emotional expression, with error bars indicating the standard error of the mean (SE). For the P100-N170 interval, Happy faces elicited the largest positive to negative amplitude variation (P100 minus N170), followed by Sad and Neutral expressions (significant differences: * *p*<0.05). For the N170-P250 interval, Sad faces showed reduced peak-to-peak amplitude variation (P250 minus N170) compared with both Neutral and Happy expressions. These results indicate differential modulation of early and intermediate ERP components as a function of emotional facial expression.

**P100: Occipital Channels (O1, Oz, O2)**

Across the occipital electrodes O1, Oz, and O2, P100 amplitude did not differ significantly between happy, neutral, and sad faces. Mean amplitudes were highly similar across conditions, and a one‑way repeated‑measures ANOVA confirmed the absence of an emotional modulation of the P100 (*F* (2,38) = 2.15, *p* = .130, *ηp*² = 0.102. Similarly, P100 latency did not differ between emotional conditions. Mean latencies were nearly identical across happy, neutral, and sad faces, and the one‑way repeated‑measures ANOVA again indicated no effect of emotion (*F*(2,38) = 1.15, *p* = .328, *ηp*² = .057;
 **Supplementary Table 1:** Mean amplitude (µV) and latency (ms) of the P100 component at occipital electrodes O1, Oz, and O2 across emotional conditions (Neutral, Sad, Happy). Values are presented as average ± standard deviation.

| **P100 Amplitude (O1, Oz, O2)** | Neutral | 7.28 ± 4.84 µV |
| --- | --- | --- |
|  | Sad | 7.03 ± 4.80 µV |
|  | Happy | 7.34 ± 4.83 µV |
| **P100 Latency (O1, Oz, O2)** | Neutral | 132 ± 10.08 ms |
|  | Sad | 132 ± 10.53 ms |
|  | Happy | 131 ± 8.59 ms |

**Faces vs. Objects Analysis**

In the faces *versus* objects analysis, the P100 component did not show statistically significant differences between conditions. Mean amplitudes were comparable (Faces: 2.87 µV; Objects: 2.52 µV), and the paired‑samples t‑test indicated no significant effect. In contrast, the N170 amplitude showed a robust stimulus‑category effect. Faces elicited a significantly more negative response (-2.30 µV) than objects (-0.39 µV), as confirmed by the paired‑samples test, *t*(19) = -6.01*, p* = 0.000009, mean difference = -1.91 µV, 95% CI [-2.57, -1.24]. Regarding latency, the N170 also differed significantly between conditions. Faces were processed more rapidly (184 ms) than objects (201 ms), with a mean difference of approximately 17 ms. This effect was statistically significant, ***t*(19) = -3.44, *p* = 0.003, 95% CI [-27.20, -0.60].** Thus, in addition to its larger amplitude, the N170 exhibited a significantly shorter latency for faces, reflecting faster and more efficient processing of facial stimuli.

**Supplementary Figure 2**: Grand‑average ERP waveforms elicited by faces and objects. The plot displays the mean potential (µV) over time (ms), with shaded areas representing the standard deviation (SD). Faces (blue) elicited a stronger negative deflection relative to objects (red), particularly around the N170 time window. Vertical dashed lines indicate the approximate latencies of the P100, N170, and P250 components. Time zero corresponds to stimulus onset.
